# Iodine Overload and Severe Hypothyroidism in Two Neonates

**DOI:** 10.4274/jcrpe.v1i6.275

**Published:** 2010-12-08

**Authors:** Selim Kurtoğlu, Leyla Akın, Mustafa Ali Akın, Dilek Çoban

**Affiliations:** 1 Erciyes University, Faculty of Medicine, Department of Pediatric Endocrinology, Kayseri, Turkey; 2 Erciyes University, Faculty of Medicine, Department of Neonatology, Kayseri, Turkey; +90 352 437 49 37selimk@erciyes.edu.trErciyes University Faculty of Medicine, Department of Pediatric Endocrinology, Kayseri, Turkey

**Keywords:** Neonate, hypothyroidism, Iodine overload

## Abstract

Iodine overload frequently leads to transient hyperthyrotropinemia or hypothyroidism, and rarely to hyperthyroidism in neonates. Iodine exposure can be prenatal, perinatal or postnatal. Herein we report two newborn infants who developed severe hypothyroidism due to iodine overload. The overloading was caused by excessive use of an iodinated antiseptic for umbilical care in the first case, and as a result of maternal exposure and through breast milk with a high iodine level in the second case. Presenting the two cases, we wanted to draw attention to these preventable causes of hypothyroidism in infants.

**Conflict of interest:**None declared.

## INTRODUCTION

Prenatal, perinatal or postnatal iodine overload can cause problems such as transient hyperthyrotropinemia, hypothyroidism or hyperthyroidism in preterm and term infants (1, 2, 3, 4, 5). Herein we report two term neonates who developed hypothyroidism due to iodine overload caused by umbilical care with iodine solution in patient No. 1, and through breast milk in patient No. 2. 

## CASE REPORT

**Case 1**

A 13-day-old male was referred to our clinic because of a high thyrotropin (TSH) level (42.9 mU/L) detected in a blood sample taken for congenital hypothyroidism screening on the fourth day of life. The baby’s birth weight was 3250 g and his length was 51 cm. History revealed that an iodine-containing antiseptic had been applied 3 times a day for umbilical care since birth. The mother had no autoimmune thyroid disease. On the 8^th^ day of life, laboratory data showed a TSH level of 120 mU/L (N: 0.5-6.5 mU/L, by IRMA) and a thyroxine (T4) level of 6.08 μg/dl (N: 8.2-17.2 μg/dl). An X-ray of the knee showed a bone maturation level of 38 gestational weeks. Thyroid ultrasonography revealed a thyroid volume of 1.1 mL (N: 0.79mL) (6). Urinary iodine level was 51 μg/dl (N:10-20 μg/dl). On his 13^th^ day of life, thyroid function tests were: Free triiodothyronin (fT3) level 1.79 pg/mL (N: 2.5-3.9 pg/mL), fT4 4.69 pg/mL (N: 9-26 pg/mL), TSH 66.11 mU/L (N: 0.5-6.5 mU/L) and thyroglobulin 2.36 ng/mL (N: 2-106 ng/mL). L-T4 treatment was started in a dose of 13 μg/kg/day. Seven days after initiation of treatment, laboratory data revealed a fT3 level of 5.19 pg/mL, a fT4 level of 27.41 pg/mL and a TSH level of 0.44 mU/L. The L-T4 dose was reduced to 6.5 μg/kg/day. 14 days later, thyroid functions were: fT3: 2.02 pg/mL, fT4:13 pg/mL and TSH: 0.22 mU/L. On the most recent visit (on the 30^th^ day of treatment), laboratory results were: fT3: 3.4 pg/mL (N: 2.5-3.9 pg/mL), fT4: 14.29 pg/mL (N:9-26 pg/mL), and TSH: 3.23 mU/L (N: 0.5-6.5 mU/L). The hypothyroidism was accepted to be due to iodine overload caused by excessive application of iodine to the umbilicus. It was decided to continue the replacement therapy in the same dose, and eventually to gradually reduce the dose and discontinue the treatment.  

**Case 2**

A 15-day-old male was referred to our clinic due to a high TSH (26.11 mU/L) and a low fT4 level (6.83 pg/mL). The baby was born by vaginal delivery and an episiotomy was applied during the delivery. The mother used an iodine antiseptic for her episiotomy incision for about 10-12 days postpartum. The baby’s knee X-ray showed a bone maturation of 40 weeks. His thyroid volume was reported as 1.04 mL measured by thyroid ultrasonography (N: 0.79mL) (6). Urinary iodine level was 41 μg/dl (N: 10-20 μg/dl). Iodine concentration of the mother’s breast milk was 30 μg/dl (N: 10-20 μg/dl). The mother did not have any autoimmune thyroid disease. The hypothyroidism in this case was attributed to iodine overload due to iodine transfer during breastfeeding. L-T4 treatment at a dosage of 8 μg/kg/day was initiated. On the follow-up visit on the 40^th^ day of life, while the baby was on the same dose of L-T4, the laboratory data were as follows: fT3: 3.23 pg/mL, fT4:9.9 pg/mL, TSH: 3.04  mU/L.

It was planned to reduce the L-T4 dose according to the results of the future thyroid function tests and to eventually discontinue the treatment. 

## DISCUSSION

Maternal intake of excessive iodine or  contrast agents and the use of drugs or antiseptics containing iodine can cause fetal and neonatal iodine overload via transplacental transport or breastfeeding.  Iodine overload in the neonate can also occur due to the use of contrast agents and iodine antiseptics in the infant during the postnatal period (1, 2, 4, 8, 9, 10). The use of iodine antiseptics during birth increases the iodine level of the cord blood by 50% in minutes and also causes iodine overload in the mother. Iodine levels in maternal urine and breast milk increase tenfold if iodine is used for episiotomy (11). Iodinated antiseptics used for umbilical care may also cause iodine overload because they are rapidly absorbed through the excessively permeable skin of the neonates. The low renal clearance of iodine, particularly in newborns with renal insufficiency, can facilitate this condition (3). Hypothyroidism due to iodine overload can develop more easily and be more severe in iodine-deficient areas (11).

Hyperthyroidism caused by iodine overload is very rare in newborns (5). However, recall of hyperthyrotropinemic infants encountered in newborn screening has led to an increase in prevalence of cases of overt hypothyroidism and this is more common if iodine overload exists (3, 7, 9, 12). Although the sensitivity threshold may differ interindividually, the fetus and newborn are both quite sensitive to iodine overload (7). The cut-off points for urinary iodine levels for overload have been reported as 16, 20 or 25 μg/dl by different authors (13, 14, 15). Generally, an iodine level above 20 μg/dl in the urine or breast milk can be accepted as iodine overload (3, 16).

When iodine overload occurs, hormone biosynthesis in the thyroid gland is blocked by an autoregulation mechanism, known as the Wolff-Chaikoff effect (17, 18). TSH-induced cyclic AMP formation, sodium-iodide symporter (NIS) expression, iodine uptake and organification decrease, while MIT/DIT ratio increases, and proteolysis decreases. This effect is transient and ceases within 25-60 hours (17). However, if iodine overloading persists, hypothyroidism becomes evident.

The management of hypothyroidism caused by iodine overload is controversial. However, it is recommended that overt hypothyroidism should be treated and followed-up (10). In many centers the treatment is continued until three years of age. Treatment can be tapered down and discontinued at an earlier age if thyroid hormone levels are elevated. It must be kept in mind that it is more important to prevent the iodine overload in both mothers and babies. Health caregivers should be educated on this subject.
